# Impact of single versus dual arterial supply on perfusion and function in finger replantation after complex hand injuries

**DOI:** 10.1038/s41598-025-85525-x

**Published:** 2025-01-25

**Authors:** Martynas Tamulevicius, Malte David Steinbach, Florian Bucher, Nadjib Dastagir, Doha Obed, Peter M. Vogt, Khaled Dastagir

**Affiliations:** Department of Plastic, Aesthetic, Hand and Reconstructive Surgery, Hannover Medical School, Carl-Neuberg-Str. 1, D-30625 Hannover, Germany

**Keywords:** Complex hand injuries, Finger injuries, Perfusion, Hand function, Disability, Comorbidities, Hypoxia, Chronic pain, Trauma

## Abstract

Finger amputations following complex hand injuries (CHI) pose a significant challenge in hand surgery due to severe tissue trauma and neurovascular damage, necessitating precise arterial repair. While restoring arterial perfusion is critical, it remains unclear whether reconstructing both proper palmar digital arteries is required for optimal outcomes. This study evaluates whether restoring one or both arteries in finger replantation after complex injuries impacts perfusion and overall outcomes. In this retrospective, cross-sectional, follow-up study, we investigated patients with finger amputations following CHI admitted to the high-volume regional hand trauma center between January 2013 and December 2020. Perfusion has been assessed using FLIR thermal imaging and laser speckle contrast analysis. Functional outcomes and quality of life scores were measured using standardized questionnaires. Sensory assessments, along with pain and grip strength measurements were also conducted. A total of 31 patients were included in the study. Thermal imaging showed a significantly higher finger surface temperature in patients with two-artery reconstruction. Laser speckle contrast analysis confirmed better perfusion, though not statistically significant. Functional and quality-of-life scores were similar across groups, except for significantly improved temperature sensation in the two-artery group. In conclusion, reconstructing both arteries in finger replantation following CHI isn’t essential for good outcomes if one artery provides adequate perfusion, but dual reconstruction may improve perfusion and temperature sensation.

## Introduction

Hand injuries are among the most frequent injuries treated in emergency departments, accounting for up to 30% of all visits^1,2^. These injuries not only result in immediate functional impairment but also carry long-term consequences for patients, including loss of dexterity, chronic pain, and reduced quality of life^3^. The National Electronic Injury Surveillance System (NEISS) data from the United States, spanning January 2009 to December 2018, shows that hand and finger injuries are the most common, with incidence rates of 264 and 450 per 100,000, respectively^4^. These findings emphasize not just the need for targeted prevention but also effective treatment protocols for hand injuries due to their high frequency and significant impact on hand function.

When multiple tissue types of the hand—such as tendons, nerves, or bones—are simultaneously injured, the condition is classified as a complex hand injury^5,6^. The age group most affected by these injuries consists of working-age men, 20–59 years old^4,5^. Such injuries pose significant challenges for both surgical repair and rehabilitation, as it is crucial to restore optimal hand function and enable working-age individuals to return to their jobs as quickly as possible^7^. Hand reconstructive surgery aims to restore function, sensation, and appearance, striving to achieve the best possible outcome after injury. The management of complex hand injuries has evolved from basic splinting or amputation in ancient times to highly specialized microsurgical procedures today^8–10^. It involves addressing multiple aspects, including soft tissue reconstruction, osteosynthesis of fractures, and repair of exposed joints and lacerated tendons^6^. Predominantly restoring blood flow through arterial and venous anastomosis and nerve function through nerve repair is essential. While some studies suggest that increased blood flow at wound sites does not always enhance wound healing or nerve regeneration, it is universally accepted that adequate perfusion to the distal structures of the complex injured hand is essential for proper wound healing^6,11^. Moreover, vascular comorbidities like diabetes or atherosclerosis are linked to an increased risk of complications after hand surgery, indicating that reduced blood flow may impair the healing process^12^.

The blood supply to each finger comes from two proper digital arteries^13^. Complex hand injuries are frequently accompanied by traumatic amputation of one or more fingers. In cases where both arteries are compromised, the resulting ischemia makes immediate reconstruction essential to prevent irreversible tissue damage^14^. Success rates for single-finger replantation vary widely, ranging from 50 to 86%, with higher success rates associated with younger patients and more experienced surgeons^15,16^. For vascular repair, single arterial anastomosis may be sufficient in certain cases, including multiple-digit replantation, as suggested by findings from individual studies^17^. In distal finger replantations (Tamai zones I and II), artery-only repair remains a viable option when vein anastomosis is not feasible, while for proximal replantations (Tamai zone III or higher), repairing at least one vein significantly increases survival rates^18^. Moreover, the functional outcomes of finger replantation are influenced not only by vascular perfusion but also by the overall condition of the patient and the nature of the injury. Factors such as the mechanism of injury (e.g., crush vs. clean-cut injuries) and the presence of nerve damage can significantly affect recovery^19,20^. Studies have shown that replantation success rates can vary widely, with estimates ranging from 57 to 95%, depending on these variables^21^. A recent retrospective study by Dastagir et al. showed that in patients with a single digital artery injury, one-vessel supply to the finger is sufficient for wound healing and functional outcomes^22^. However, it remains unclear whether reconstructing only one artery in an amputated finger of a complexly injured hand is sufficient to achieve comparable functional outcomes. This retrospective cohort study aimed to evaluate how outcomes in complex hand injuries with single-digit amputation differ based on whether one or both arteries were repaired, using a combination of patient-reported outcomes, clinical assessments, and advanced imaging to assess functional recovery. 

## Methods

### Study design

**Fig. 1:**
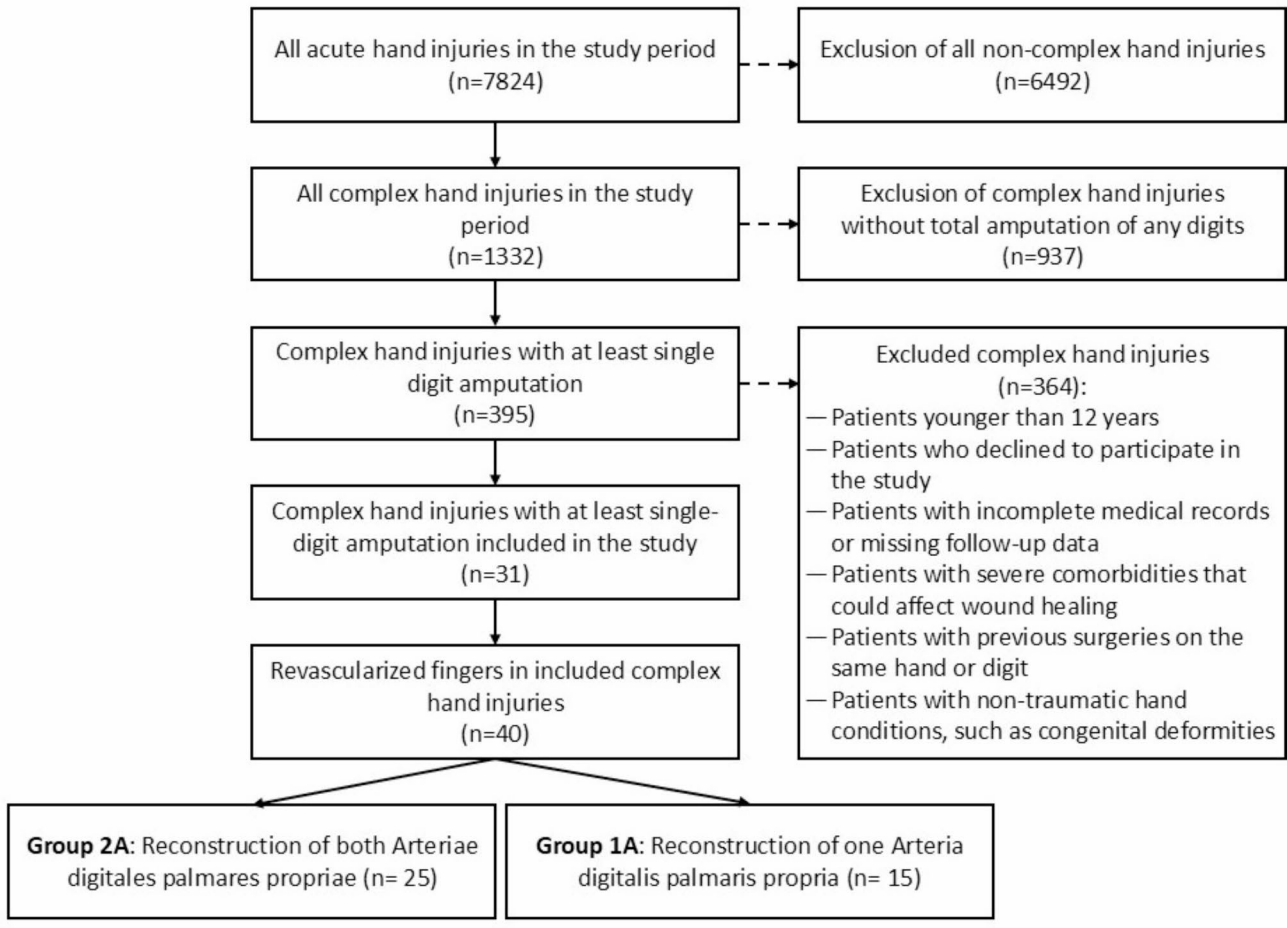
Flowchart of the study.

In this retrospective, cross-sectional, follow-up study we investigated patients who presented with complex hand injuries, including at least a single-digit amputation, at the Federation of European Societies for Surgery of the Hand (FESSH) certified hand trauma and replantation center between January 2013 and December 2020. The patients were divided into two cohorts: those who underwent arterial reconstruction and achieved a two-vessel blood supply (Group 2 A) and those who received reconstruction of only one artery due to extensive tissue damage (Group 1 A). Patients younger than 12 years were excluded from the study, as it was assumed they would not be able to provide reliable answers to the clinical questionnaires during the examinations and interviews. Likewise, all patients who declined to participate in the study were excluded. Of the 395 invited patients, 31 (7.85%) agreed to participate. Due to the COVID-19 pandemic, 13 of the 31 patients (41.94%) opted for a telephone interview instead of an in-person examination. The remaining 18 participants (58.06%) chose to attend an in-person examination (s. Figure 1).

The study was conducted in accordance with the ethical guidelines of the Declaration of Helsinki and received approval from the local ethics committee (No. 8778_BO_K_2018). All participants volunteered for the study, with consent forms signed and dated by both the patients and the surgeons. The decision to restore a single-vessel blood supply was made by the surgeon. Arterial reconstruction was performed using either direct anastomosis or interpositional vein grafts, depending on the extent of arterial damage and the feasibility of achieving a tension-free repair. Vein grafts were harvested from the volar aspect of the distal forearm when arterial defects were too extensive for direct anastomosis. In all cases, the priority was to ensure an adequate arterial anastomosis on at least one side to maintain perfusion. Situations leading to single-artery reconstruction included significant substance defects and tissue trauma that did not allow adequate coverage of the neurovascular bundle on one side or very distal arterial damage (e.g., fingertip injuries) where anastomosis out of trauma zone was not possible. In such cases, the decision to reconstruct only one artery prioritized achieving a tension-free and adequately perfused anastomosis on the remaining viable side. Dual arterial reconstruction was pursued when feasible without compromising the overall integrity of the neurovascular structures. At least two veins were repaired in order to restore venous outflow, ensure adequate drainage and minimize the risk of congestion. In all cases, nerve coaptation was performed. The arterial flow of the digital arteries was confirmed using Doppler ultrasound at follow-up.

Patient-reported outcomes were evaluated using multiple standardized questionnaires. The Disabilities of the Arm, Shoulder, and Hand (DASH) questionnaire asked patients to score their physical function and symptoms following finger reconstruction on a scale of 0 to 100, with 0 representing no restriction and 100 representing maximum disability. The Patient-Rated Wrist/Hand Evaluation (PRWE-G) assessed pain and functional difficulties, with scores ranging from 0 (no pain or difficulty) to 100 (worst pain or difficulty). The EQ-5D-5 L questionnaire was used to assess overall quality of life in five dimensions: mobility, self-care, usual activities, pain/discomfort, and anxiety/depression. Additionally, the EuroQol visual analog scale (EQ-VAS) was used to assess self-reported health on a scale from 0 to 100, where 0 represents the poorest health status and 100 is the best.

Clinical assessments included two-point discrimination (2PD) using a caliper, grip strength using a Jamar^®^ hand dynamometer, and cold sensitivity evaluation. For grip strength, each patient performed three maximal grip trials on each hand, and the average was calculated to account for individual variability. Cold sensitivity was assessed by asking patients about discomfort in cold environments. Temperature sensitivity was evaluated by having patients touch warm and cold objects with both the affected and unaffected hands and compare sensations. To measure circulatory perfusion, FLIR thermal imaging (FLIR ONE^®^ Pro, © Teledyne FLIR LLC, Wilsonville, Oregon, USA) and laser speckle contrast analysis (LASCA) (PeriCam PSI System, Perimed AB, Järfälla, Sweden) were used to capture and compare blood flow between the injured and uninjured hands. FLIR thermal imaging detects radiation of the objects in the long-wave or mid-wave infrared spectrum, converting it into a visual thermal image. A LASCA camera works by illuminating tissue with a laser, capturing the fluctuating speckle pattern created by scattered light, and analyzing the speckle contrast to generate perfusion images that reflect blood flow. These imaging techniques provided precise measurements of temperature and blood flow, which are critical for assessing vascular function in reconstructed hands. The FLIR and LASCA ratios were used to normalize the quantitative measurements of the injured finger against the corresponding finger on the non-injured hand, accounting for baseline variability, and were calculated by dividing the perfusion value of the injured finger at the fingertip by the perfusion value of the same finger at the fingertip on the non-injured hand. To gain a deeper understanding of the patient’s reported pain symptoms, the Visual Analog Scale (VAS) was used. Patients were asked to rate their pain at rest (VAS-R) and under load (VAS-L) on a scale of 1 to 10 during both in-person physical examinations and telephone interviews. A score of 1 indicates no pain, while 10 represents the worst imaginable pain.

## Estimation of sample size

To determine the appropriate sample size for this study, a Type I error (α) of 0.05 and a power (1-β) of 0.80 were applied. The sample size calculation was conducted using Fisher’s exact test, which is suitable for small sample sizes and categorical data. The calculation was performed with G*Power software (Düsseldorf, Germany), and it was determined that a minimum of 14 patients would be required to achieve sufficient statistical power.

### Statistical analysis

Values are presented as the mean ± standard deviation for metric data, with ranges (minimum and maximum) provided. Ordinal data are expressed as absolute numbers and percentages. All data were tested for normal distribution. Statistical analysis was conducted using SPSS (IBM Corp., Armonk, NY, USA) and GraphPad Prism version 9.5.1 (GraphPad Software for Windows, San Diego, California, USA). A P-value of less than 0.05 was considered statistically significant. For initial comparisons between the two study groups, nominal data were analyzed using Pearson’s chi-squared test. If more than 20% of expected frequencies were less than 5, Fisher’s exact test was used for two groups, or the Fisher-Freeman-Halton exact test for more than two groups. For pairwise comparisons of normally distributed metric data, an unpaired t-test was applied. For comparisons with more than two normally distributed groups, one-way ANOVA was used, followed by Bonferroni-corrected post-hoc tests if significant differences were found. Non-normally distributed metric data were compared using the Mann-Whitney U test for two groups and the Kruskal-Wallis H test for more than two groups. Correlations between metric variables were analyzed using Pearson’s correlation for normally distributed data and Spearman’s correlation for non-normally distributed data. Linear regression analysis was performed following correlation analysis to assess the relationship between continuous variables.

## Results

### Patient and injury demographics

Most of the patients were male (*n* = 28, 90.3%). The mean age of the patients was 58.97 ± 16.99 years (range 16–85 years). In 14 patients (45.2%), the left hand was affected by the complex hand injury, and in 17 participants (54.8%), the right hand was affected. Of these, 30 identified as right-handed (96.8%) and one as left-handed (3.2%). In 16 participants (51.6%), the dominant hand was affected, while in 15 (48.4%), the non-dominant hand was injured. Of the 75 injured fingers, 40 (53.33%) underwent revascularization. Most patients (24, 77.4%) had a single complexly injured finger, while 7 patients (22.6%) had multiple fingers affected. The causes of injury could be divided into four groups: avulsion injuries (9 fingers, 22.5%), guillotine-type injuries (4 fingers, 10%), circular saw injuries (25 fingers, 62.5%), and crush injuries (2 fingers, 5%). All finger injuries were avascular and in 30 patients (75%) there remains a partial connection, such as through skin or tendons. Moreover, 95% of patients had at least one digital nerve injury. In 72.5% of avascular fingers, both digital nerves were injured. On average, 0.95 nerves were injured per avascular finger.

In 25 fingers (62.5%), both proper palmar digital arteries (2 A) were reconstructed, while in 15 fingers (37.5%), only one artery (1 A) was restored. Thirteen affected fingers were thumbs (32.5%), 12 were index fingers (30%), 7 were middle fingers (17.5%), 6 were ring fingers (15%), and 2 were small fingers (5%). On average, patients underwent 1.87 ± 0.92 surgeries (range 1–4) related to the hand injury. After the initial surgery, patients stayed in-hospital an average of 8.6 ± 4.19 days (range 3–17). At the time of evaluation, the mean follow-up time since the first surgery was 3.89 ± 2.69 years (range 0.23–8.53). Relevant medical diagnoses and pre-existing conditions, such as diabetes mellitus, dyslipidemia, peripheral arterial disease, arterial hypertension, nicotine abuse, and alcohol abuse, were extracted from the hospital information system. Sixteen patients (51.6%) had at least one of these conditions, with the following frequencies: diabetes mellitus (19.4%), peripheral arterial disease (6.5%), arterial hypertension (38.7%), dyslipidemia (12.9%), and nicotine abuse (16.1%). No cases of alcohol abuse were recorded. There were no statistically significant differences in patients` and injury demographics between the groups (s. Table 1).

**Table 1:** Comparative analysis of patients` demographics between the groups.

		Group 1 A	Group 2 A	*P* value
Sex	Male	14 (100%)	14 (82.4%)	0.232
	Female	0 (0.0%)	3 (17.6%)	
Age (years)		61,07 ± 15,69	57,24 ± 18,27	0.679
BMI (kg/m²)		28,25 ± 3,40	26,36 ± 4,46	0.215
Injured finger	D1	8 (53.3%)	5 (20.0%)	0.214
	D2	3 (20.0%)	9 (36.0%)	
	D3	1 (6.7%)	6 (24.0%)	
	D4	2 (13.3%)	4 (16.0%)	
	D5	1 (6.7%)	1 (4.0%)	
Type of injury	Avulsion	1 (6.7%)	8 (32.0%)	0.102
	Guillotine-type	1 (6.7%)	3 (12.0%)	
	Circular saw	13 (86.7%)	12 (48.0%)	
	Crush	0 (0.0%)	2 (8.0%)	
Level of injury	Proximal phalanx	5 (33.3%)	10 (40.0%)	0.767
	Proximal interphalangeal joint	3 (20%)	4 (16.0%)	
	Middle phalanx	3 (20%)	8 (32.0%)	
	Distal interphalangeal joint	3 (20%)	2 (8.0%)	
	Distal phalanx	1 (6.7%)	1 (4.0%)	
Type of amputation	Total	4 (26.7%)	6 (24.0%)	0.927
	Subtotal	11 (73.3%)	19 (76.0%)	
Follow-up time (years)		5.02 ± 3.09	2.96 ± 1.95	0.440
Average number of surgeries		2.14 ± 1.03	1.65 ± 0.77	0.186
Duration of inpatient treatment (days)		9.00 ± 4.51	8.29 ± 4.01	0.706
Tendon injuries	At least one	14 (93.3%)	21 (84.0%)	0.633
	None	1 (6.7%)	4 (16.0%)	
Nerve injuries	One side	4 (26.7%)	5 (20.0%)	0.730
	Both sides	11 (73.7%)	18 (72.0%)	
	None	0 (0.0%)	2 (8.0%)	
Bone injuries	Yes	11 (73.7%)	20 (80.0%)	0.705
	No	4 (26.7%)	5 (20.0%)	
Comorbidities	Diabetes	3 (21.4%)	3 (17.6%)	1.000
	Peripheral arterial disease	1 (7.1%)	1 (5.9%)	
	Arterial hypertension	5 (35.7%)	7 (41.2%)	
	Nicotine abuse	2 (14.3%)	3 (17.6%)	
	Dyslipidemia	2 (14.3%)	2 (11.8%)	
	Alcohol abuse	0 (0.0%)	0 (0.0%)	
Long-term medication	Lipid-lowering agents	2 (14.3%)	2 (11.8%)	1.000
	Antihypertensive drugs	5 (35.7%)	6 (35.2%)	
	Antidiabetic drugs	3 (21.4%)	2 (11.8%)	
	Anticoagulants / Aspirin	5 (35.7%)	4 (23.5%)	

### Perfusion analysis

**Fig. 2:**
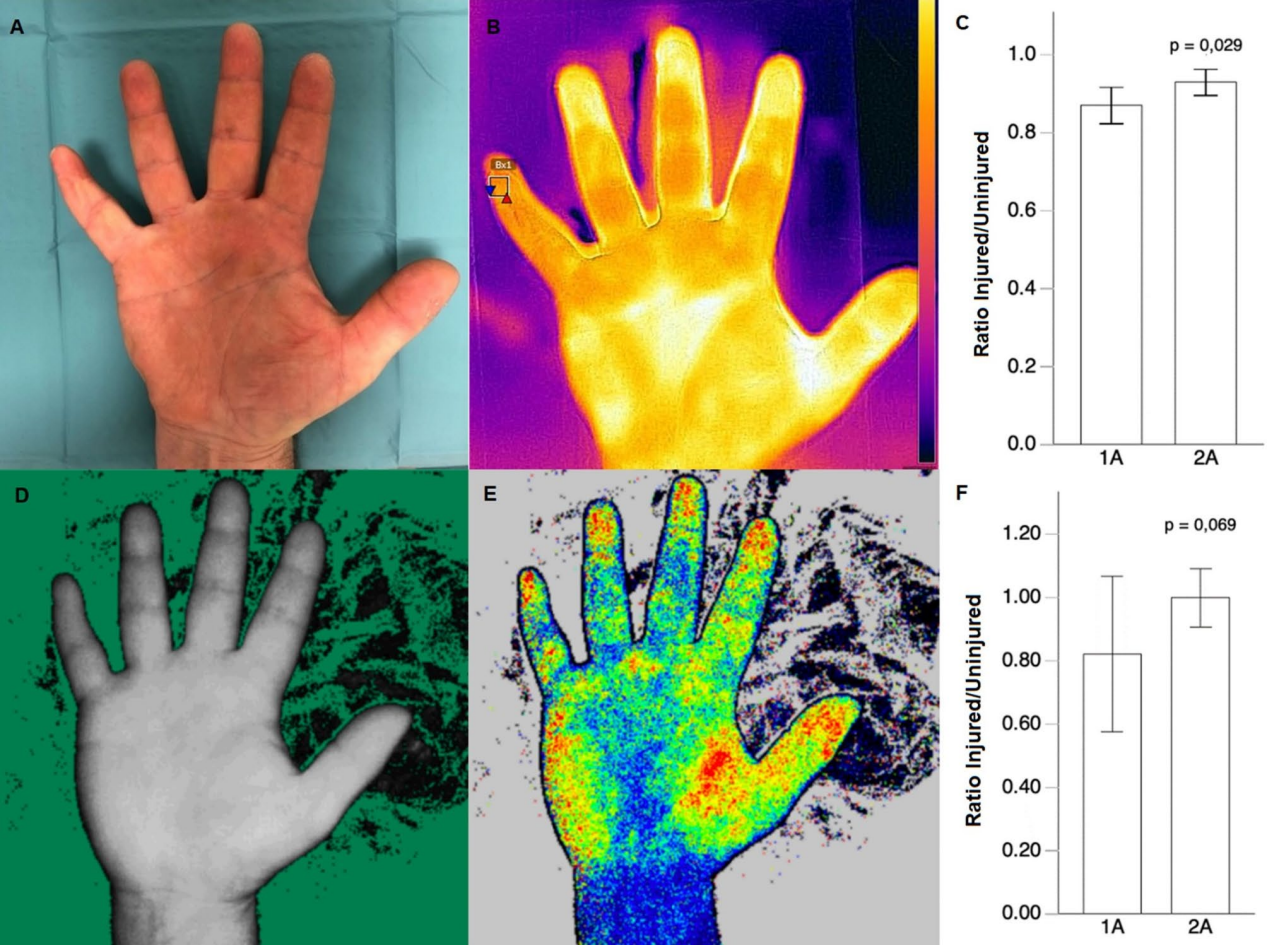
Representative images of FLIR imaging (**A**,**B**) and LASCA imaging (**D**,**E**). The circulation ratio between the injured hand and the patient’s uninjured hand: FLIR imaging (**C**) and LASCA imaging (**F**). Data are presented as mean ± SEM.

The perfusion of the revascularized fingers in complex hand injuries was measured using a FLIR thermal imaging camera and a LASCA camera. In the 1 A group, the mean FLIR ratio between the complex injured and contralateral uninjured hand was 0.87 ± 0.06 (range 0.801–0.958). In the 2 A group, the mean value was 0.92 ± 0.056 (range 0.839–1.013). A statistically significant improvement in perfusion parameters was identified in the 2 A group (*p* = 0.029). The mean LASCA ratio in the 1 A group was 0.82 ± 0.27 (range 0.51–1.19), while in the 2 A group, the mean was 1 ± 0.17 (range 0.74–1.36). However, no statistically significant difference between groups was found (*p* = 0.069) s. Figure 2). Further analysis of the FLIR and LASCA ratios between the injured and uninjured hands, based on injury type and injury level, showed no statistically significant differences, with *p* = 0.679 and *p* = 0.388 for injury type, and *p* = 0.685 and *p* = 0.107 for injury level, respectively. Additionally, we analyzed the potential association between follow-up time and perfusion. A trend toward better perfusion was observed in patients with longer follow-up, although it did not reach statistical significance (FLIR thermal imaging: ρ = 0.342, *p* = 0.129; LASCA: ρ = 0.186, *p* = 0.408).

### Sensation analysis

In the clinical analysis of thermoreception, 6 out of 25 examined fingers (24%) were unable to clinically distinguish between cold and warm, indicating a complete loss of thermoreception. In 7 fingers (28%), patients could clinically differentiate between cold and warm, but reported issues in daily activities compared to the healthy side, such as delayed perception of high temperatures. In 12 fingers (48%), reliable thermoreception was observed without any limitations in daily activities compared to the uninjured side. A comparison of the percentages revealed a significant difference in thermoreception between the 1 A and 2 A study groups (*p* < 0.001) (s. Table 2). Moreover, a potential association with cold intolerance was examined, but no statistically significant relationship between the groups was found (*p* = 0.311). A two-point discrimination measurement was also conducted. The average nerve measurement in the 1 A group was 16.3 ± 5,73 mm, while in the 2 A group, it was 15.36 ± 5,51 mm. No statistically significant difference was found between these values (*p* = 0.866).

**Table 2:** Distribution of thermoreception results in the 1 A and 2 a groups.

	Group 1 A	Group 2 A	*P* value
Loss of thermoreception	4 (50.0%)	2 (11.8%)	*p* < 0.001
Reduced thermoreception	2 (25.0%)	5 (29.4%)	
Normal Thermoreception	2 (25.0%)	10 (58.8%)	

### Patient-reported outcomes and grip strength analysis

The patient-reported outcomes showed comparable functional results after complex hand injuries. The DASH scores, PRWE scores, EQ-5D-5 L scores and EQ-VAS scores showed no statistically significant differences (*p* = 0.581, *p* = 0.562, *p* = 0.305 and *p* = 0.112 respectively). Moreover, there were no statistically significant differences between the two groups in perceived pain both at rest and under load (s. Table 3). The clinical examination revealed comparable grip strength outcomes between the groups. The Jamar^®^ ratio, expressed as strength percentage compared to the unaffected side, had a mean of 57.2 ± 35.58% in the 1 A group and 52.99 ± 37.11% in the 2 A group.

**Table 3:** Patient-reported outcomes.

	Group 1 A	Group 2 A	*P* value
DASH-Score	18,50 ± 20,43	13,70 ± 14,28	0,581
PRWE-Score	20,12 ± 19,39	14,07 ± 12,38	0,562
EQ-5D-5 L	0,75 ± 0,26	0,64 ± 0,30	0,305
EQ VAS	69,67 ± 20,75	57,05 ± 27,01	0,112
NAS at rest	1,27 ± 0,84	0,88 ± 0,70	0,361
NAS under load	2,93 ± 2,15	2,40 ± 1,85	0,978

## Discussion

Complex hand injuries can significantly impact critical everyday activities like writing, eating, lifting, and manipulating different objects. In addition to the significant psychosocial burden and long-term physical disability, many patients are often unable to return to their previous occupations following the injury^23–25^. In the context of complex hand injuries, finger amputations often occur, affecting various anatomical structures^6,26^. In such cases restoring adequate perfusion is highly important, as blood circulation in the injured tissue is directly associated with adequate wound healing^27–29^. Moreover, in complex hand injuries, it’s not only the wounds that require adequate perfusion, but also other damaged tissues like injured nerves, bones, and tendons^30–32^. Like any healing wound, there is an important risk of infection. An impaired blood supply can increase the risk of wound infection by compromising the immune system’s ability to eliminate bacteria, as it significantly impairs the function of immune cells^33^. Any disruptions in the healing process can delay rehabilitation, potentially leading to worse outcomes and reduced hand function.

We observed differences in blood flow between the 1 A and 2 A groups in finger amputations following complex hand injuries, with higher FLIR and LASCA ratios in the 2 A group. The difference in FLIR imaging was statistically significant. A previous study by Dastagir et al., which focused on isolated arterial injuries, found no difference in perfusion between fingers with single-vessel and two-vessel supply. Collateral circulation from the contralateral side was proposed as the explanation for these comparable results^22^. However, this mechanism does not seem applicable to complex hand injuries. In our study, the larger tissue defects and more extensive surgical trauma in complex injuries may have led to the destruction of potential collateral pathways. Research has shown that collateral vessel formation is critical for maintaining blood flow in the extremities when primary circulation fails^34^. In complex hand injuries, extensive soft tissue damage may impair both neovascularization and the development of collateral circulation, explaining the differences in blood flow observed between the groups. Additionally, a trend was observed between the follow-up time and the LASCA and FLIR measurements. This suggests that collateralization may continue to occur even years after the finger amputation, potentially leading to improved overall perfusion. Multiple analyses of cerebral perfusion indicate that collateralization is particularly prevalent in chronic vascular occlusions and may continue over an extended period, not just during the acute healing phase when tissue perfusion is compromised^35,36^. Furthermore, recurrent episodes of reduced perfusion can serve as triggers for neovascularization^37^. In contrast, it is well-established that increased perfusion, driven by angiogenesis and capillarization, is especially prominent during the early stages of wound healing^38^. In humans, wound healing typically culminates with scar formation after approximately one year^39^. During this time, the observed increase in perfusion is primarily linked to the ongoing inflammatory response, characterized by leukocyte infiltration and elevated oxygen demand in the tissue^40^. However, further research is required to elucidate the relationship between perfusion and time since initial treatment to gain a deeper understanding of collateralization processes.

Even though no significant differences in perfusion parameters were observed in the replanted fingers, we believe that reconstructing both arteries should always be prioritized whenever feasible. Dual arterial reconstruction reduces the risk of significant finger loss due to potential perfusion disturbances, such as thrombosis in the remaining artery. This risk is especially critical in cases where the boundaries of the trauma zone are unclear. In cases of minimal crush injuries to both arteries, we recommend reconstructing at least one artery using a venous graft, as sufficient perfusion can often be restored even after thrombectomy. When thrombosis is present in an artery without an actual continuity defect, we suggest excising the injured segment due to the thrombogenicity of the damaged intima^41^. For finger amputations, it is essential to debride the vessels both proximally and distally until the intima remains intact. However, visual confirmation of intima integrity can sometimes be challenging, necessitating careful surgical judgment. Vein grafting with two anastomoses, while technically more complex and potentially thrombogenic^42,43^, has not been shown in large clinical studies to worsen outcomes^44,45^. Even in fingertip amputations, vein grafting has been shown to improve survival rates^43,46^. Moreover, digital artery reconstruction using interposition vein grafts has shown promising results in treating ischemic conditions of the hand, such as secondary Raynaud’s phenomenon, where it effectively improved ischemic pain and ulceration^47^. Regarding venous reconstruction, we routinely perform at least two venous anastomoses to restore adequate drainage and reduce the risk of congestion. This strategy is supported by current literature, which demonstrates higher success rates (64%) in replantation procedures with multiple venous repairs compared to those with none or a single repair (46%)^48^.

Regarding the patient-reported questionnaires on hand functionality and quality of life, no significant differences were found between the groups. The results of the DASH, PRWE, EQ-5D-5 L, and EQ-VAS questionnaires showed comparable outcomes in terms of patient-relevant factors, such as limitations in the use of the affected hand in daily life and overall quality of life. This suggests that despite better blood flow in the 2 A group, there is no significant advantage in hand functionality for patients. Similar observations were made in a study by Sadaba et al., which found that although blood flow in the hand decreased after the removal of the radial artery for myocardial revascularization, there was no short-term impairment in hand function (65). However, there remains a risk of necrosis or gangrene if sufficient arterial perfusion from the other side is not maintained. In a retrospective review by Valentine et al., eight patients over five years developed hand ischemia after radial artery cannulation for hemodynamic monitoring, with a mean duration of ischemia of 3 ± 2 days and an incidence of 1 in 1000^49^. It is important to note that the hand function assessed by the DASH and PRWE questionnaires, as well as the quality of life and mood parameters evaluated by the EQ-5D-5 L and EQ-VAS, are not solely influenced by whether an amputated finger is supplied by one or two arteries. Complex hand injuries vary significantly between individuals, particularly in terms of size, depth, and the affected tissues. The functional outcome for the patient also depends on which finger is injured. For instance, the loss of the thumb’s distal phalanx accounts for a 10% reduction in overall functional capacity, as does the complete loss of the small finger^50^. Therefore, it is clear that some complex finger injuries may be compensated in terms of overall hand function, which may explain the lack of significant differences in questionnaires that assess global hand function or quality of life. These findings support the compensation theory, as both groups objectively showed comparable results in grip strength measurements using the Jamar^®^ dynamometer. Unfortunately, there are no alternative questionnaires specifically designed to evaluate the outcomes of complex hand injuries more accurately.

In the management of finger amputations, restoring both adequate perfusion and nerve coaptation is critical, as nerves are essential not only for daily function but also for wound healing^51^. In some cases, nerve coaptation may even take priority over arterial repair, especially when one artery remains intact^22^. This highlights the interconnectedness of perfusion and nerve regeneration^52^. Our study found no significant differences in two-point discrimination (2PD) between the 1 A and 2 A groups, with averages of 16.27 mm and 15.36 mm, respectively. These values are higher than in comparable studies, possibly due to greater tissue damage in complex hand injuries. This may indicate that nerve regeneration is affected by the destruction of small vessels, like the vasa nervorum, rather than by the presence of a single intact artery. In terms of thermoreception, a higher percentage of patients in the 2 A group retained functional thermoreception, with more patients in the 1 A group showing a complete loss of sensation. This difference may be related to better blood flow in the 2 A group, as perfusion differences were evident in FLIR and LASCA imaging. This improved thermoreception represents the only significant functional benefit observed in the two-artery group, highlighting the importance of optimizing perfusion to maintain adequate thermoreception. This is particularly relevant for individuals whose professions demand fine tactile precision (e.g., surgeons, musicians, or jewelers) or those exposed to cold environments.

There are some limitations in our study. Finger amputations following complex hand injuries vary significantly in size and affected structures, complicating the establishment of a homogeneous patient cohort. Functional outcomes, particularly those reported by patients, can differ substantially based on the specific injured finger, with the thumb being more functionally significant than the small finger. The retrospective design also introduces limitations, including correlations that may not imply causation and the potential for selection bias due to the single-center setting. A multicenter approach would help mitigate this bias. Additionally, variations in follow-up duration may have influenced perfusion outcomes, as previously discussed. The retrospective nature of the study also restricted our ability to control the accuracy and consistency of previously recorded surgical and patient history data. Despite these limitations, the study offers valuable insights into the effects of various treatments on functional outcomes and quality of life in patients with finger amputations following complex hand injuries.

Our study demonstrates that in finger amputations following complex hand injuries, there is a significant difference in perfusion between fingers with a single-vessel supply and those with a two-vessel supply. However, better blood flow does not automatically lead to better functional outcomes for the patient. Two-point discrimination (2PD), cold intolerance, and grip strength showed comparable results in both groups. Similarly, the DASH, PRWE, EQ-5D-5 L, and EQ-VAS questionnaire outcomes showed no significant differences between the groups. Only temperature perception was significantly more functional in the two-vessel group than in the single-vessel group. Our data suggests that improved perfusion has no significant effect on key functional outcomes, which may have a greater impact on the patient’s hand function and overall quality of life. However, we believe restoring adequate perfusion is crucial for the smooth healing of all injured structures in complex hand injuries, therefore if a two-vessel supply can be established without major challenges, it should be considered to avoid potential complications such as spasms or thromboembolic occlusions, and also to promote optimal wound healing and reduce infection risk. Future studies should explore whether improved perfusion can significantly reduce postoperative complications.

## Data Availability

The data supporting the findings of this study are available from the corresponding author upon reasonable request. Due to privacy concerns, the dataset is not publicly archived. Access to the data will be provided in compliance with institutional guidelines and applicable regulations to ensure individual privacy is protected.
